# Identification of active small-molecule modulators targeting the novel immune checkpoint VISTA

**DOI:** 10.1186/s12865-021-00446-4

**Published:** 2021-08-11

**Authors:** Ting-ting Li, Jing-wei Jiang, Chen-xin Qie, Chun-xiao Xuan, Xin-lei Hu, Wan-mei Liu, Wen-ting Chen, Jun Liu

**Affiliations:** Jiangsu Key Lab of Drug Screening, China Pharmaceutical University, Nanjing, 210009 China

**Keywords:** VISTA, Small-molecule modulator, Antagonist, Homology modeling, Virtual screening

## Abstract

**Background:**

Cancer immunotherapy has gained increasing popularity as a novel approach to treat cancer. A member of the B7 family, V-domain immunoglobulin suppressor of T-cell activation (VISTA) is a novel immune checkpoint that regulates a broad spectrum of immune responses. VISTA is an acidic pH-selective ligand for P-selectin glycoprotein ligand-1(PSGL-1). CA-170, a first-in-class small-molecule dual antagonist of VISTA/PD-L1, was collaboratively developed by Aurigene Discovery Technologies Limited and Curis, Inc. It is currently in Phase I clinical trial.

**Results:**

In this study, we develop homology modeling for the VISTA 3D structure and subsequent virtual screening for VISTA small-molecule hit ligands. Visualization of the binding postures of docked ligands with the VISTA protein indicates that some small molecular compounds target VISTA. The ability of antagonist to disrupt immune checkpoint VISTA pathways was investigated though functional studies in vitro.

**Conclusions:**

Affinity active molecule for VISTA was obtained through virtual screening, and the antagonist compound activity to VISTA was assayed in cellular level. We reported a small molecule with high VISTA affinity as antagonist, providing ideas for development VISTA-targeted small molecule compound in cancer immunotherapy.

**Supplementary Information:**

The online version contains supplementary material available at 10.1186/s12865-021-00446-4.

## Background

Immune checkpoints protect peripheral tissues from damage during immune responses. Immune checkpoint blockade has become one of the most promising approaches to activating antitumor immunity [1, 2]. Immune checkpoint inhibitors may release the brake on the immune system, allowing it to target cancer cells. Antibodies targeting immune checkpoint cytotoxic T lymphocyte antigen-4 (CTLA-4) and programmed death protein 1 (PD-1)/programmed cell death ligand 1 (PD-L1) have been broadly applied in the clinic and have yielded promising results as immunotherapy for diseases such as late-stage metastatic melanoma and nonsmall cell lung cancer (NSCLC) [3–8]. Several ongoing trials with immune checkpoints inhibitors (PD-1/PD-L1) might bring new insights for role of immunotherapy for patients with stages I to III NSCLC [9].Vaccination with dendritic cells (DCs) or DCs/cytokine-induced killer (CIK) cells has shown limited success in the treatment of patients with advanced non-small-cell lung cancer [10]. Immunotherapy, mainly vaccine-based (aiming to activate the host immune system to induce human immune response to tumour-specific antigens) did not make people live longer. But it seemed that those who were given vaccine-based immunotherapy may have experienced, on average, more side effects. Therefore, reseachers are testing new, more promising immunotherapy drugs (e.g. checkpoint inhibitors) [11].

However, the clinical application of antibody-based immunotherapy has encountered a number of problems, including a low response rate (20–30%) [4], the inconvenience of intravenous administration, immune-related adverse events (irAEs) and high cost for patients. In recent years, scientists have been dedicated to identifying additional immune checkpoint pathways and orally available small molecules to overcome these flaws.

Among the expanding list of immune checkpoints, V-domain immunoglobulin suppressor of T-cell activation (VISTA) [12] has been indicated as an important regulator of the immune system. VISTA is a type I transmembrane protein and bears features of both the B7 and CD28 families of immunoregulatory molecules. Murine VISTA extracellular Ig-V domain with 136-aa linked to a 23-aa stalk segment has 76% homology with the human protein [13]. Within hematopoietic cells, CD11b^high^ myeloid cells, e.g., monocytes, conventional dendritic cells (DCs), and macrophages can overexpress VISTA [14]. VISTA regulates a broad spectrum of immune responses [14–20]. VISTA is reported as an acidic pH-selective ligand for PSGL-1 recnetly [21]. Studies of multiple tumor models, autoimmune disease models and clinical samples have demonstrated a pivotal regulatory role of VISTA on the immune system and its potential as a therapeutic or combinational drug target.

The feasibility of targeting VISTA for cancer treatment is manifested by findings indicating high VISTA expression on tumor cells in approximately 20% of NSCLC specimens [22]. Clinical studies have shown that VISTA expression is upregulated in gastric and oral squamous carcinoma [23, 24]; it is also increased after ipilimumab therapy in patients with prostate cancer [25–28]. Furthermore, previous studies have shown that VISTA is highly expressed in immune cell subsets from human pancreatic cancer patients [28].

The VISTA antibody drug trial was terminated in the clinical phase [29]. Noelle et al. first identified a peptide of VISTA antagonists through phage display. The peptide antagonist enhanced T cell proliferation in vitro and significantly enhanced antitumor immunity [30]. A small-molecule inhibitor of VISTA/PD-L1 (CA-170, the compound structure has not yet been disclosed)-collaboratively developed by Aurigene Discovery Technologies Limited and Curis, Inc-can restore the IFN-γ production inhibited by soluble VISTA upon induction of human peripheral blood mononuclear cells (PBMCs) with anti-CD3 and anti-CD28 antibodies [31, 32]. Currently, compound CA-170 is undergoing Phase I clinical trial for advanced tumors and lymphoma (NCT02812875) [33]. CA-170 exhibits potent activity when tested in assays to rescue lymphocyte proliferation and effector functions inhibited by PD-L1, PD-L2, or VISTA/PD-1H proteins. In a panel of functional assays, CA-170 showed selectivity against other immune checkpoint pathways including CTLA4, LAG3, and BTLA. These non-clinical data provided a strong rationale for the clinical development of CA-170 [34–36].

In addition to being a promising target for cancer treatment, VISTA is also a key regulator of several types of autoimmune inflammatory diseases due to its multifaceted role in modulating both innate and adaptive immune responses. Antagonistic or agonistic agents can conceivably modulate VISTA and its interacting partners (VSIG8 and VSIG3) [37, 38], which will greatly benefit the treatment of autoimmune and inflammatory diseases.

In this study, we sought to discover and develop small-molecule antagonists targeting VISTA. Compounds were screened by molecular docking and virtual screening. Then, protein binding activity assays and a cell activity evaluation were performed to provide insights into the development of small-molecule antagonists of VISTA.

## Methods

### Homology modeling of the VISTA three-dimensional structure

Homology modeling of the VISTA 3D structure was as reported previously [39]. The extracellular domain without the signal peptide of human (162 amino acids, UniProt: Q9H7M9) and murine(159 amino acids, UniProt: Q9D659) VISTA protein were submitted to the I-TASSER online server [40–42] to generated the three-dimensional model [39].

### Virtual screening of small-molecule ligands docked with VISTA

The construction of 3D models and methods for virtual screening are reported previously [39]. Firstly, the ligand-binding pockets was predicted,and the docked ligands with a binding energy lower than − 6.0 kcal/mol were considered as the candidate murine VISTA ligands. Then, the best 20 candidate ligands were selected to verify their protein–ligand interaction following the subsequent experiments (ELISA) in Additional file 1 (Additional file 1: Table S1). Lastly, the lowest Kd values of the hit ligand detected by ELISA was applied for the VISTA-targeted in vitro experiment.

### Visualization of docked ligands with the VISTA protein

As reported previously [39], the amino acid residues of the murine VISTA proteins involved in the protein–ligand interaction were regarded as potential interactive residues if they close to the hit ligands (≤ 1 Å).

### Microscale thermophoresis (MST) experiment

Following the manufacturer’s instructions provided in the Monolith NT™ Protein Labeling Kit RED-NHS (NanoTemper Technologies GmbH), the murine VISTA-ECD was labeled accordingly. A 16-step serial dilution of compound 6809-0223 in the repective binding buffer was prepared, equal volume of labeled protein was added and mixed by pipetting. Then, the mixtures were incubated at room temperature for 30 min in the dark and filled into standard capillaries.

### Animals

Mice (C57BL/6) were purchased from Beijing Vital River Laboratory Animal Technology Co., Ltd. All animal work were followed by the Laboratory Animal Management Committee of Jiangsu Province and approved by the ethics committee of China Pharmaceutical University. Mice were kept on a 12-h light/dark cycle with food and water provided ad libitum.

### Murine CD4^+^ and CD8^+^ T cell activation assays

According to the manufacturer’s instructions (Stem Cell), the murine CD4^+^ and CD8^+^ cells were isolated from the spleen of C57BL/6. Purified CD4^+^ and CD8^+^ cells were stimulated by murine CD3(clone 2C11) and either VISTA-Ig or control-Ig with different ratio which coated in 96-well flat-bottom plates. After 48 h, the cell supernatants was harvested for cytokine secretion assay. For T-cell proliferation assay,purified CD4^+^ and CD8^+^ cells were labeled by CFSE (5-(and-6)-carboxyfluorescein diacetate, succinimidyl ester) dye (Biolegend) for 20 min at 37 °C and 5% CO_2_ according to manufacturer’s protocol. CFSE-labeled T cells were plated into the pre-coated 96-well flat-bottom plates described above.T cell proliferation was assessed at 72 h by flow cytometer.

### Cytometric bead assay (CBA) for CD8^+^ T cells

CD8^+^ T cells were isolated as described above, and a density of 1 × 10^5^ cells/well were plated into the 96-well flat-bottom plates coated with anti-mouse CD3 (clone 17A2, Biolegend) at 2.5 μg/mL together with 5 μg/mL of murine VISTA-ECD protein. The compound 6809-0223 diluted into indicated concentrations was added to the culture medium. The cytokine levels in the cell supernatant collected at 48 h were analyzed using a BD Cytometric Bead Array (CBA) kit according to the manufacturer’s instructions.

### Statistical analysis

The data are analyzed and expressed as the mean ± SD unless indicated otherwise. Statistically significant differences was determined by unpaired Student’s t-test. A value of *P* < 0.05 was considered significant at the 95% confidence level.

## Results

### Virtual screening for VISTA small-molecule hit ligand

The criteria for selection of candidate compounds has been described previously [41]. The 20 candidate ligands were selected for the subsequent experimental protein–ligand interaction verification by ELISA. The binding rates of murine VISTA-ECD with the compounds selected by virtual screening were evaluated with the ELISA assay (Additional file 1: Table S1). Twenty candidate compounds (binding energy less than − 6 kcal/mol) were selected for experimental verification. The predicted binding energie for one hit ligand (6809-0223) showed good binding affinity for murine VISTA. The binding energie for the ligand (6809-0223) was − 9.4 kcal/mol. In the subsequent experiments, we verified its biological effect on VISTA.

**Fig. 1:**
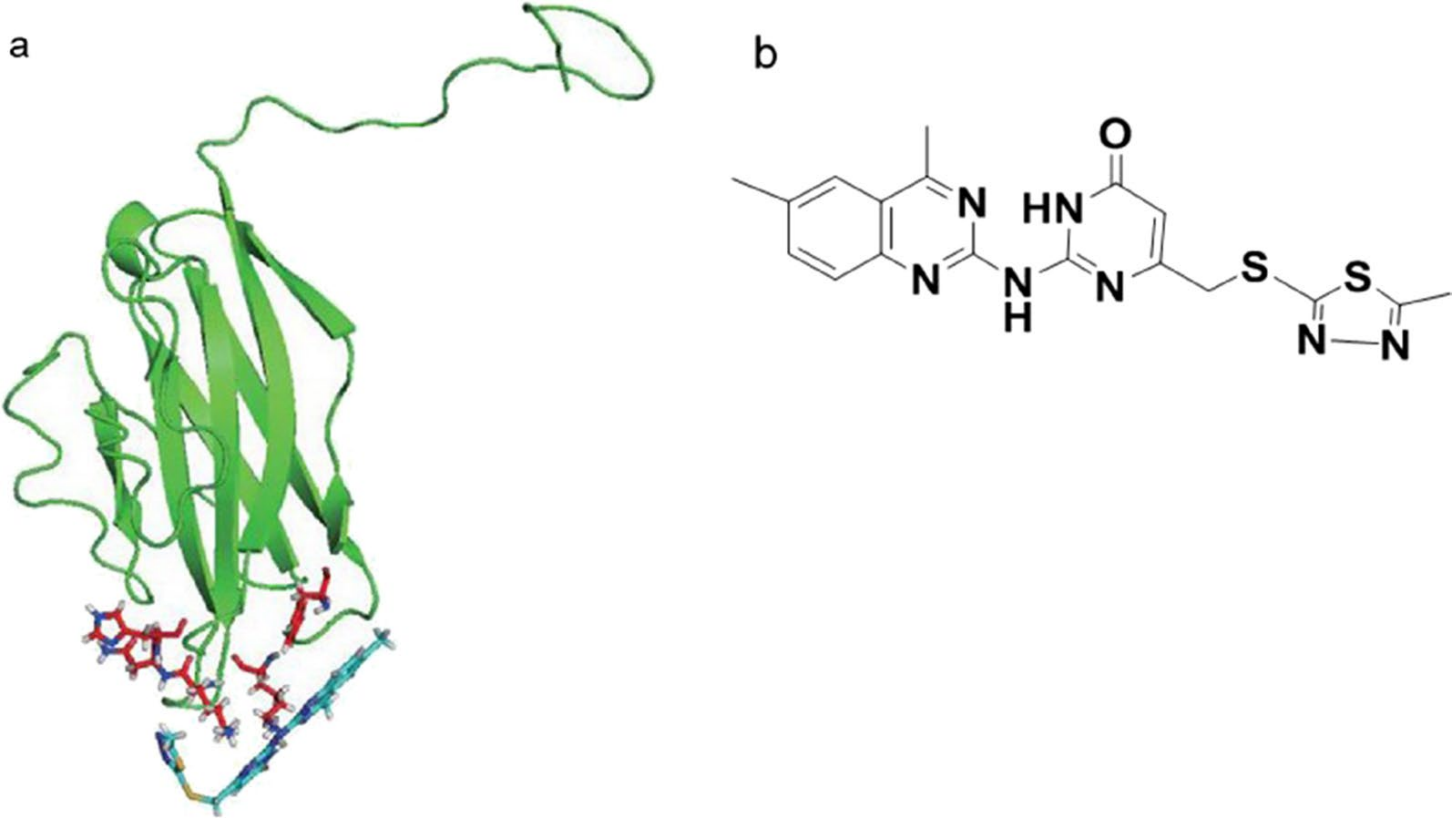
**a** Antagonist ligands (light blue) interacting with local residues of the murine VISTA protein (green). Residues on the VISTA protein interacting with antagonist ligands are highlighted in red. **a** Shows the whole VISTA protein extracellular domain and the docked positions of the ligand (6809-0223). **b** Structure of compound 6809-0223

The protein–ligand interaction visualized with the Pymol software was displayed (Fig. 1).The docked position of the ligand (6809-0223) (2 loop areas: 58–62 aa and 150–157 aa) was showed in Fig. 1. The overall docked parameter of this protein–ligand interaction was summarized in Table 1.

**Table 1:** One hit ligand showing significant agonist/antagonist effect

Macromolecule	Hit ligand	Hydrogen bonds	Potential hydrophobic interactions	Predicted binding energy (kcal/mol)
Murine VISTA	6809-0223	NA	PHE33, LYS62, LEU150, LYS151, ASN152	− 9.4

**Fig. 2:**
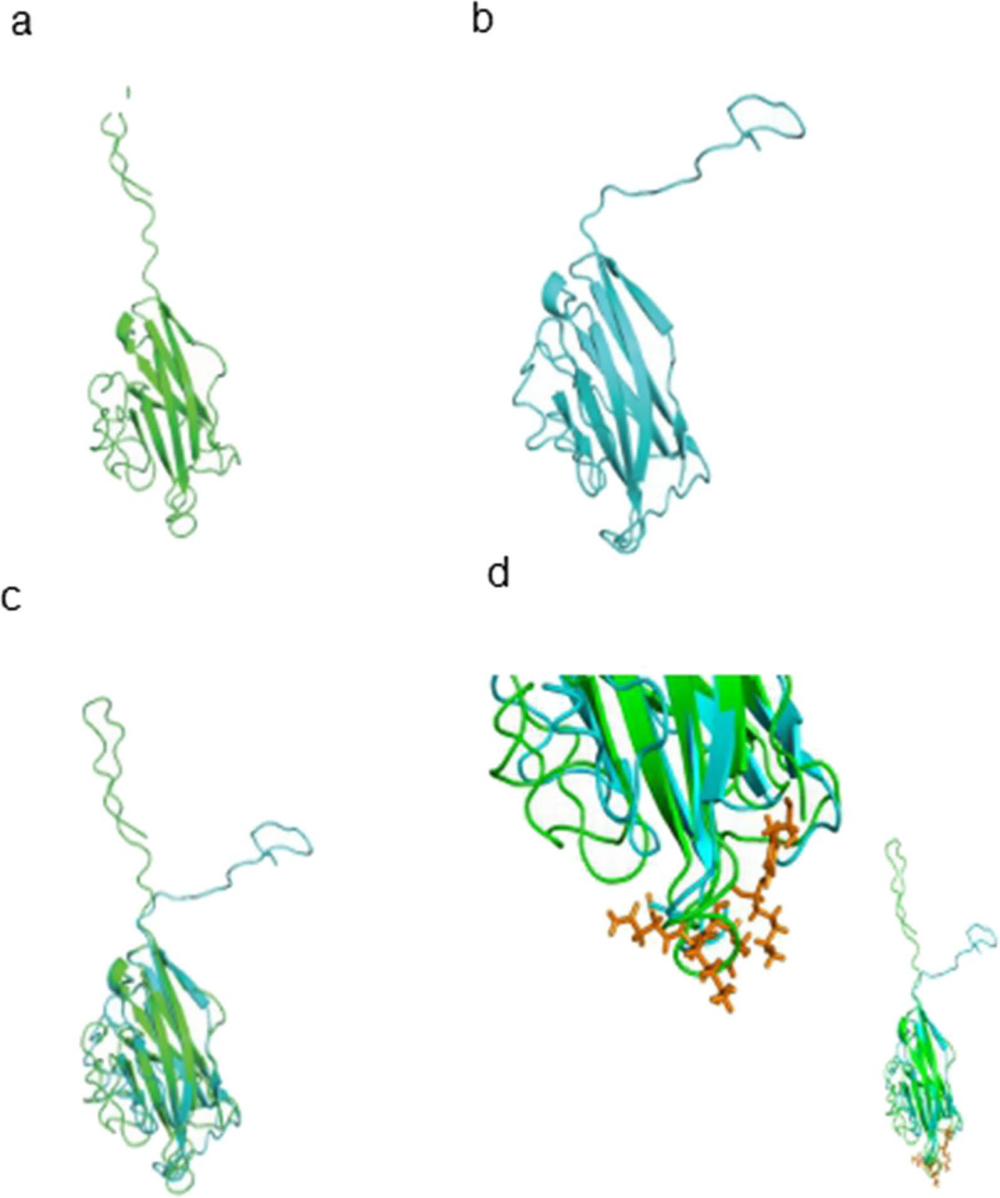
The 3D structural comparison between the human and murine VISTA proteins and their ligand-interacting residues. **a** The human VISTA protein extracellular domain (green); **b** The murine VISTA protein extracellular domain (light blue); **c** Aligned human VISTA protein (green) and murine VISTA protein (light blue); **d** Ligand interacting residues: murine VISTA antagonist interacting residues (orange), 3D structural alignment of the whole human/murine VISTA protein extracellular domain (bottom right)

The murine VISTA protein extracellular domain (159 amino acids, Fig. 2b) is slightly shorter than that of the human VISTA protein (162 amino acids, Fig. 2a). Both VISTA proteins had highly similar structures based on the 3D structural alignment (Fig. 2c). The antagonist-interacting residues for the murine VISTA proteins are located in close proximity in the 3D structure (Fig. 2d).

### The Kd value of the compound for the VISTA-ECD protein

**Fig. 3:**
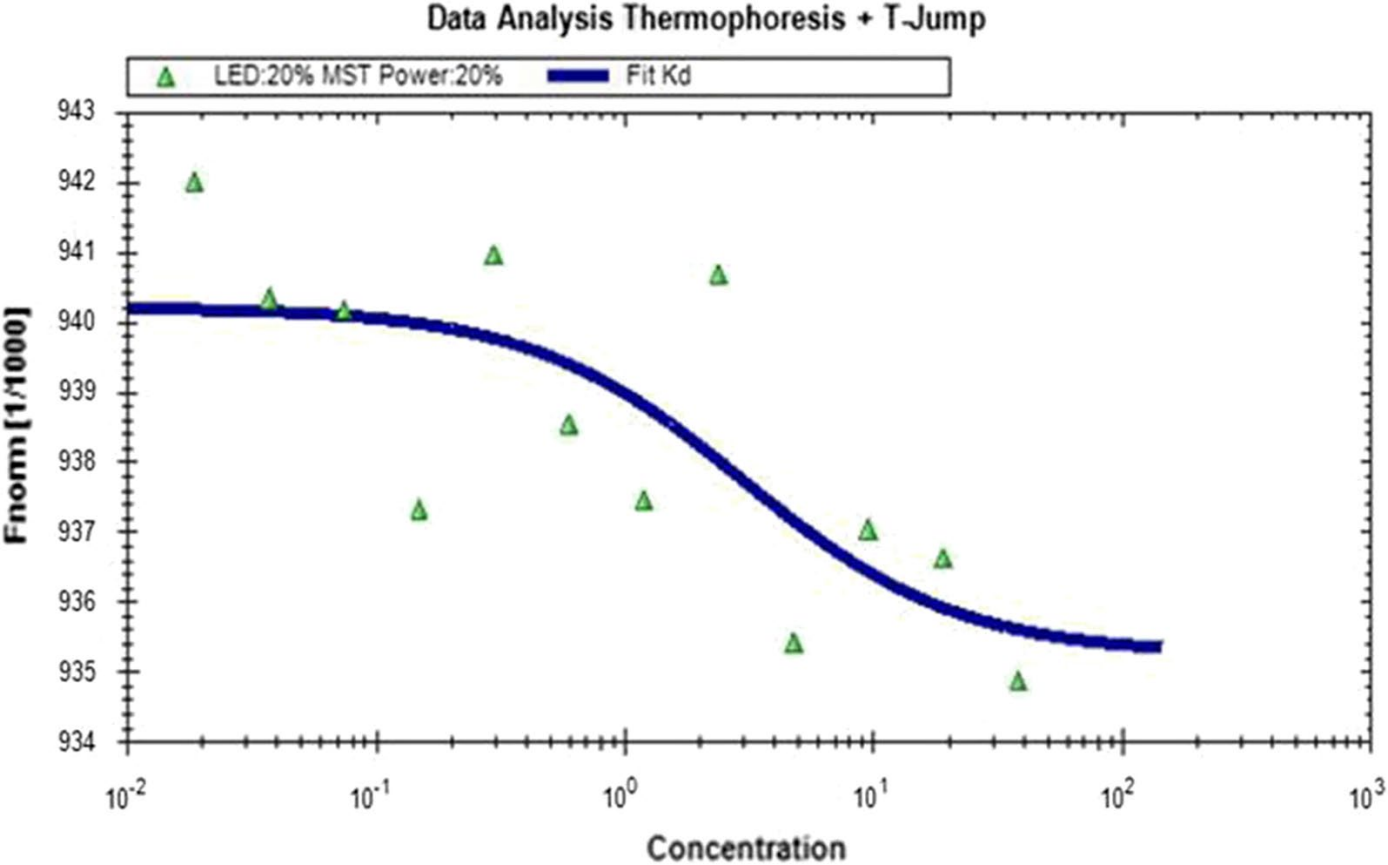
Binding affinity was evaluated between the murine VISTA-ECD protein and compound 6809-0223 by the MST experiment. All results were repeated three times with consistent results

The Kd value of compound 6809-0223 for murine VISTA-ECD was measured by MST and calculated as 0.647 ± 0.0387 μM (Fig. 3). Based on the Kd value, the binding affinity of the compounds for mVISTA was relatively weak and was at the micromolar level. The hits identified based on this approach will be a good starting point for further structural optimization in the future.

### Murine VISTA antagonist 6809-0223 increases IL-2 secretion by CD4^+^ and CD8^+^ T cells

**Fig. 4:**
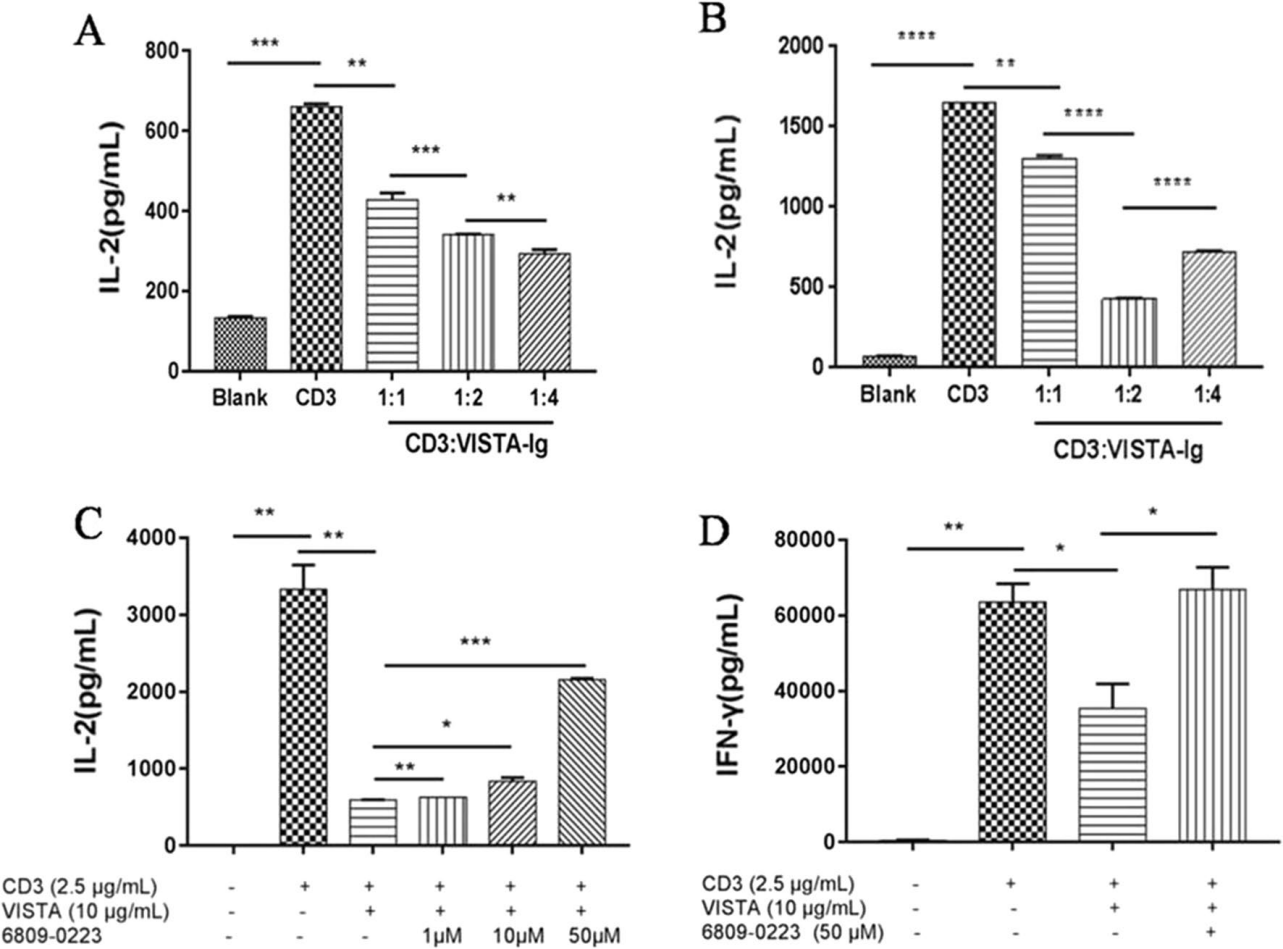
The murine VISTA protein inhibited cytokine production in T cells. **a** CD4^+^ T cells (1 × 10^5^ cells/well) were stimulated with anti-CD3 (2C11) at 2.5 μg/mL and murine VISTA at ratios of 1:1, 1:2, and 1:4. Culture supernatants were collected at 48 h. IL-2 production was analyzed by ELISA. **b** CD8^+^ T cells (1 × 10^5^ cells/well) were stimulated with anti-CD3 (2C11) at 2.5 μg/mL and murine VISTA at ratios of 1:1, 1:2, and 1:4. Culture supernatants were collected at 48 h. IL-2 production was analyzed by ELISA. **c** Purified CD4^+^ T cells (1 × 10^5^ cells/well) were stimulated with anti-CD3 (2.5 μg/mL) and the murine VISTA protein as indicated. Culture supernatants were collected after 48 h. IL-2 production was analyzed by ELISA. **d** Bulk purified CD8^+^ T cells (1 × 10^5^ cells/well) were stimulated with anti-CD3 (2.5 μg/mL) and the murine VISTA protein (5 μg/mL). Culture supernatants were collected at 48 h. IFN-γ production was analyzed by ELISA. *P < 0.05, **P < 0.01, ***P < 0.001, ****P < 0.0001. All results were repeated three times with consistent results (n = 3)

Murine T cells were plated in a 96-well plate (1 × 10^5^ cells/well) and then stimulated with a CD3 antibody (2.5 μg/mL) with or without the murine VISTA-Fc protein (2.5 μg/mL, 5 μg/mL, or 10 μg/mL). As the concentration of VISTA increased, the inhibitory effect of murine VISTA-Fc on the secretion of IL-2 by CD4^+^ and CD8^+^ T cells was enhanced (Fig. 4a, b). Compound 6809-0223 was added to the cells, and IL-2 was measured by ELISA. The results showed that compound 6809-0223 increased IL-2 secretion by CD4^+^ and IFN-γ secretion by CD8^+^ T cells (Fig. 4c, d).

### Compound 6809-0223 increased murine CD4^+^ T cell proliferation

**Fig. 5:**
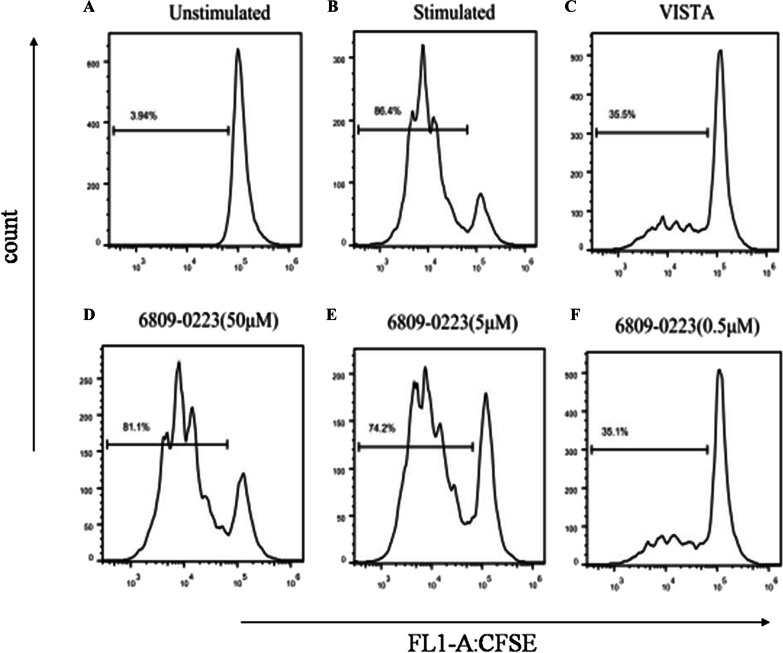
Compound 6809-0223 increases CD4^+^ T cell proliferation. CFSE-labeled cells were stimulated with anti-CD3 (2C11) at 2.5 μg/mL and murine VISTA-Fc at 5 μg/mL. Each well was seeded with 1 × 10^5^ cells/well. Cell supernatants were collected for flow cytometry at 72 h. Compound 6809-0223 promoted CD4^+^ T cell proliferation. All results were repeated three times with consistent results (n = 3)

CD4^+^ T cell proliferation was analyzed at 72 h by examining the CFSE profiles. CD4^+^ T cell proliferation was increased after compound 6809-0223 administration (Fig. 5).

### Compound 6809-0223 increased cytokine production in CD8^+^ T cells based on the CBA assay.

**Fig. 6:**
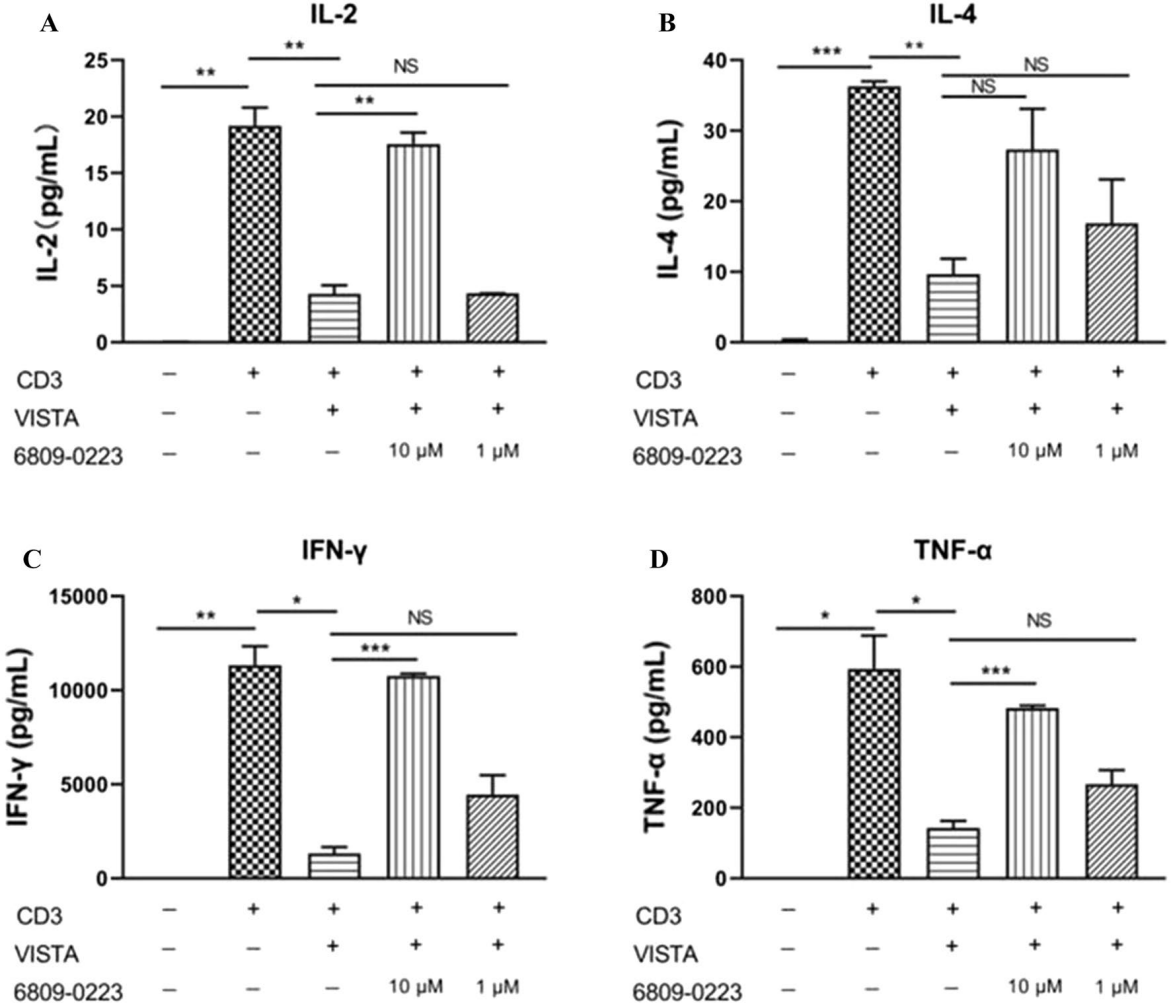
The activity of compound 6809-0223 was detected by CBA in CD8^+^ T cells. Cells (1 × 10^5^ cells/well) were incubated with an immobilized anti-mouse CD3 antibody (2.5 μg/mL) and mouse VISTA-ECD at 5 μg/mL, and compound 6809-0223 was added as indicated. The levels of **a** IL-2, **b** IL-4, **c** IFN-γ, **d** TNF-α in the cell culture supernatants were measured at 48 h with Cytometric Bead Array (CBA) Kits (n = 3)

CD8^+^ T cells were stimulated with anti-mouse CD3 antibodies, and mouse VISTA-ECD was added as indicated. The CBA results showed that production of the IL-2, IL-4, IFN-γ and TNF-α cytokines was decreased by murine VISTA-Fc. Whereas, IL-2, IL-4, IFN-γ and TNF-α production was increased by the addition of compound 6809-0223 (Fig. 6). The results showed that compound 6809-0223 could increase the secretion of certain cytokines, although the mechanism is still under investigation.

## Discussion

As reported, therapeutic antibodies of immune checkpoints possess high binding affinity for their target protein. However, its low response rate, immune-related toxicities and high cost remain unsolved problems that warrant effort to discover additional immune pathways or find alternatives to antibody-based treatment. As an alternative to monoclonal antibodies, small-molecule modulators are expected to overcome some of the aforementioned disadvantages. Discovery and development of small-molecule drugs for immune checkpoints is a promising and challenging prospect. At present, immunologic checkpoint inhibitor (ICI) have remarkable clinical efficacy in the treatment of cancers, however, the breakdown of immune escape causes a variety of immune-related adverse events, such as pneumonitis, rash and pruritus [43–46]. And this point need further investigation.

We homologously reconstructed the VISTA protein 3D structure using the protein amino acid sequence. Approximately 130,000 chemical compounds from our compound library were virtually screened using the VISTA protein 3D models. After the preliminary screening, potential compounds were tested for VISTA protein binding affinity by ELISA, and the compounds corresponding to the top three test results were selected. These selected compounds were used to adjust the predicted binding position on the VISTA protein for the second round of virtual compound screening, and the candidate molecules were repeatedly subjected to a protein affinity test until the screening results reached a plateau. The final obtained compounds are candidate compounds for verification. Although the amino acid sequence of human VISTA is slightly different from that of murine VISTA, their 3D models are very similar when structurally aligned (RMSD, root mean square deviation < 1 Å).

The predicted Mlog *P* value of the VISTA binding compound is 2.3–2.8. Combined with cellular bioassay, the compound 6809-0223 was considered lead compound for VISTA antagonist. ExpiCHO cells were used to express the murine VISTA extracellular domain proteins and obtain high purity murine VISTA proteins by affinity chromatography in Additional file 1 (Additional file 1: Figure S1 and S2). The ELISA screening model was established, and potential compounds from the virtual screen were tested for their murine VISTA protein binding affinity. To the best of our knowledge, this study is the first time to screen and identify VISTA modulators through a eukaryotic expression method. The affinity of the active compound for VISTA was further evaluated using MST experiments. We found that compound 6809-0223 had high affinity for the murine VISTA protein with Kd = 0.647 ± 0.0387 μM (Fig. 3). Compound 6809-0223 increased IL-2 secretion by CD4^+^ and CD8^+^ T cells and increased CD4^+^ T cell proliferation. Besides, the expression patterns of CD4 and CD8 in tumor tissues can be a potential option for further investigation. Their correlation with survival data need to be further studied.

In this study, the compound affinity for VISTA was relatively weak, and the activity of the molecule was in the submicromolar range. Therefore, the hit identified based on this approach is a good starting point for future optimization. The crystal structure of VISTA and the compound/VISTA complex are undergoing.

## Conclusions

**Fig. 7:**
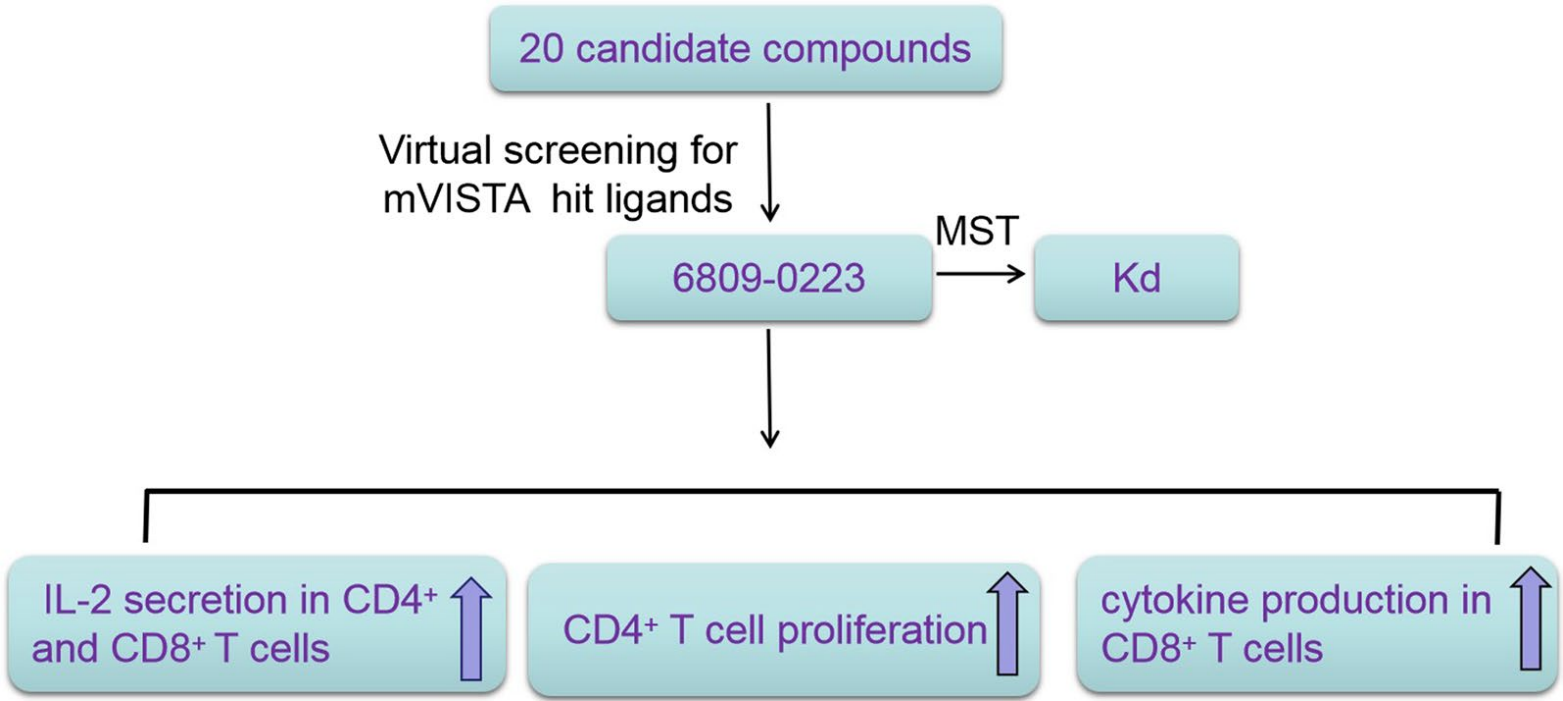
Schematic diagram of the experimental design

In summary, affinity active molecule for VISTA was obtained through virtual screening, and the antagonist compound activity to VISTA was assayed in cellular level (Fig. 7). We reported a small molecule with high VISTA affinity as antagonist, providing ideas for development VISTA-targeted small molecule compound in cancer immunotherapy.

## Supplementary Information

**Additional file 1**.** Figure S1**. Murine VISTA-Fc expression and purification. (a) Protein expression in the cell supernatant gradually increased after transfection. (b) The protein was purified from the cell supernatant using Protein A resin. The murine VISTA-Fc protein was detected by Western blotting. (c) The purity of the recombinant VISTA-Fc protein was determined by SDS-PAGE.** Figure S2**. Murine VISTA-His expression and purification. (a) Protein expression in the cell supernatants as detected by Western blotting after transfection. (b) The protein was purified from the cell supernatant using Ni Sepharose^TM^ 6 Fast Flow resin. The murine VISTA-his10 protein was detected by Western blotting. (c) The purity of the recombinant VISTA-His protein was determined by SDS-PAGE.** Table S1**. The binding rate of murine VISTA with compounds by ELISA assay.The absorbance was read at 450 nm (OD_450_) and 570nm(OD_570_).The absorbance at 570nm can be subtracted from the absorbance at 450nm. Binding rates(%) = (OD_compound_-OD_protein_)/OD_protein_ × 100%.

## Data Availability

All data in the article can be requested from the corresponding author.

## Abbreviations

CBA: cytometric bead assay
CFSE: 5- (and 6)-carboxyfluorescein diacetate succinimidyl ester
MST: microscale thermophoresis
VISTA: V-domain immunoglobulin suppressor of T-cell activation
