# Supplementary material for: Identification of active small-molecule modulators targeting the novel immune checkpoint VISTA
Source: BMC Immunol. 2021 Aug 11;22:55. doi: 10.1186/s12865-021-00446-4 (PMC8359099; doi:10.1186/s12865-021-00446-4)
Supplement: Supplementary file 1 — Additional file 1. Figure S1. Murine VISTA-Fc expression and purification. (a) Protein expression in the cell supernatant gradually increased after transfection. (b) The protein was purified from the cell supernatant using Protein A resin. The murine VISTA-Fc protein was detected by Western blotting. (c) The purity of the recombinant VISTA-Fc protein was determined by SDS-PAGE. Figure S2. Murine VISTA-His expression and purification. (a) Protein expression in the cell supernatants as detected by Western blotting after transfection. (b) The protein was purified from the cell supernatant using Ni SepharoseTM 6 Fast Flow resin. The murine VISTA-his10 protein was detected by Western blotting. (c) The purity of the recombinant VISTA-His protein was determined by SDS-PAGE. Table S1. The binding rate of murine VISTA with compounds by ELISA assay.The absorbance was read at 450 nm (OD450) and 570nm(OD570).The absorbance at 570nm can be subtracted from the absorbance at 450nm. Binding rates(%) = (ODcompound-ODprotein)/ODprotein × 100%. [file 12865_2021_446_MOESM1_ESM.doc]

**Identification of active small-molecule modulators targeting the novel immune checkpoint VISTA**

Ting-ting Li^#^, Jing-wei Jiang^#^, Chen-xin Qie^#^, Chun-xiao Xuan, Xin-lei Hu, Wan-mei Liu, Wen-ting Chen, Jun Liu^*^

*Jiangsu key lab of Drug Screening, China Pharmaceutical University, Nanjing, 210009, China*

*# These authors contribute equally to this work*

*Corresponding author: Jun Liu, PhD, E-mail: junliu@cpu.edu.cn, fax number: +86-025-83271142, telephone number: +86-025-83271043.

**Supplemental Methods and Materials**

**VISTA expression and purification.**

The murine VISTA extracellular domain (amino acids 33-191) was cloned into the pFUSE-mIgG2A-Fc expression plasmid, which was a gift from Lily Wang’s lab. The recombinant murine VISTA protein (amino acids 1-191) with 10 histidines was cloned into the pcDNA3.1 vector. VISTA-Fc and VISTA-His were produced by the ExpiCHO expression system according to the manufacturer’s instructions (Gibco). The recombinant VISTA-Fc protein was purified from the cell culture supernatant using protein Agarose (GenScript). Murine VISTA-his was purified from the cell supernatant using Ni Sepharose^TM^ 6 Fast Flow resin (GE Healthcare). The proteins were desalted into PBS using 10 KD MWCO spin columns (Millipore) and verified by blotting.

**ELISA screening model**

The purified murine VISTA-Fc extracellular domain fusion protein was diluted to 0.0982 μg/mL with coating buffer, and 100 μL of the protein solution was added to each well and incubated overnight at 4°C. The next day, the coated protein solution was discarded, and the plate was washed three times with PBST. After blocking at room temperature for 2 hours, the blocking solution was discarded, and the plate was washed 5 times with 0.01% PBST. The screening sample solution was mixed with an antibody at a 1:9 ratio (final compound concentration of 10 μg/mL; final primary antibody concentration was 0.5 μg/mLand incubated at room temperature for 2 hours. The reaction solution was discarded, and the plate was washed with PBST. A secondary anti-sheep HRP antibody was added and incubated for 1 h at room temperature, and then the plate was washed with PBST. The TMB color solution was added, and the plate was incubated in the dark for 9 min. Then, stop solution was added, and the absorbance was measured at 450 nm.

**Supplemental Results**

**Expression and purification of the murine VISTA-ECD proteins**

The murine VISTA-ECD protein was desalted into PBS and detected by Coomassie blue staining for purity assessment. We examined the purified fractions and found that mVISTA-Fc was only present in the eluted fractions (Supple. Fig. S1). We used a Protein A column to purify the murine VISTA-Fc (mVISTA-Fc) protein and used it for the subsequent ELISA screening experiments and cell activity evaluation. The murine VISTA-His protein was expressed from transected cell culture medium on the second day. The protein significantly accumulated with a prolonged transfection time. The cell culture supernatant was collected, and the recombinant proteins were purified using Ni Sepharose^TM^ 6 Fast Flow resin. We found that the murine VISTA-ECD-his protein was eluted at an imidazole concentration of 250 mM by Western blotting identification. Subsequently, we collected the 250 mM imidazole elution fractions and concentrated by Amicon Ultra-15 ultrafiltration (Merck). The protein was desalted into PBS, and the purity was evaluated by SDS-PAGE (Suppl. Fig. S2).

**Suppl. Fig S1a**

**
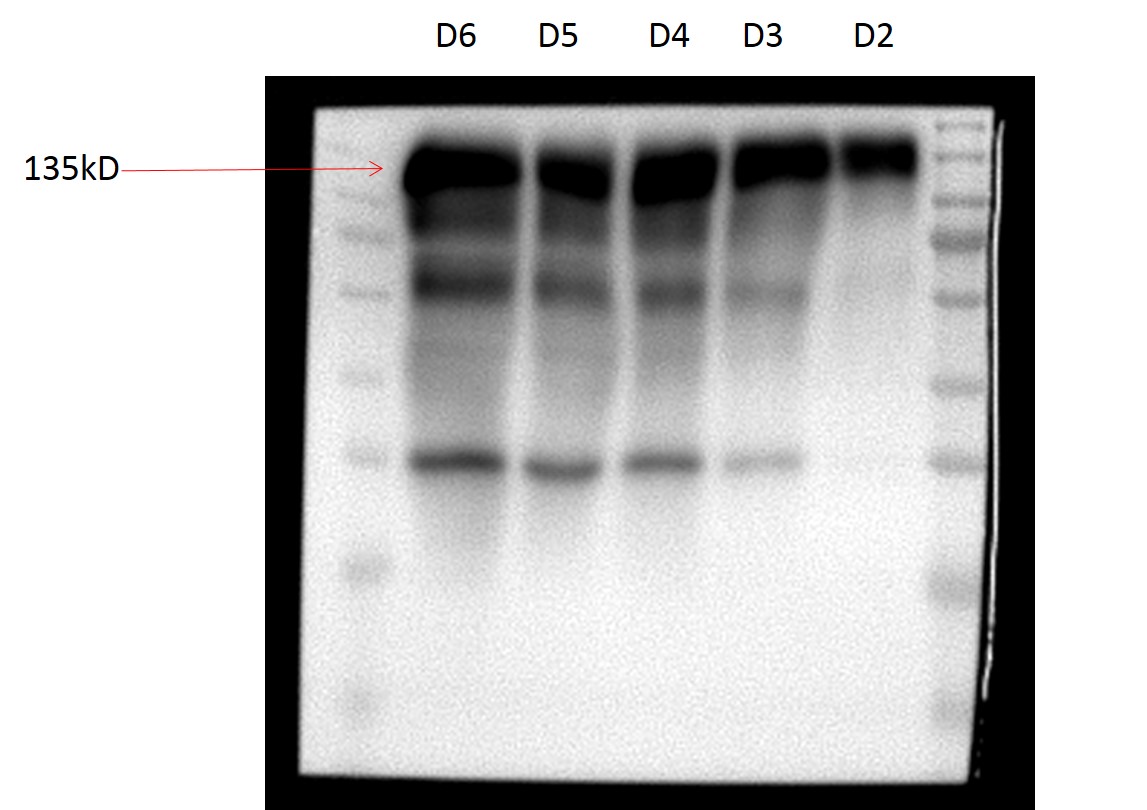
**

**Suppl. Fig S1b**

**
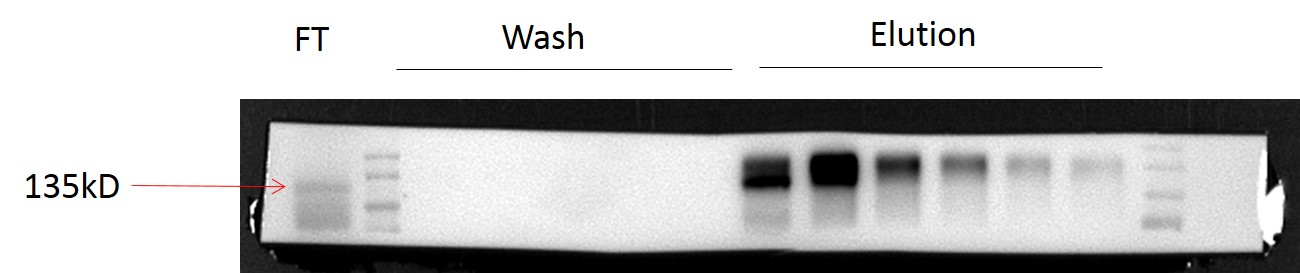
**

**Suppl. Fig S1c**

**
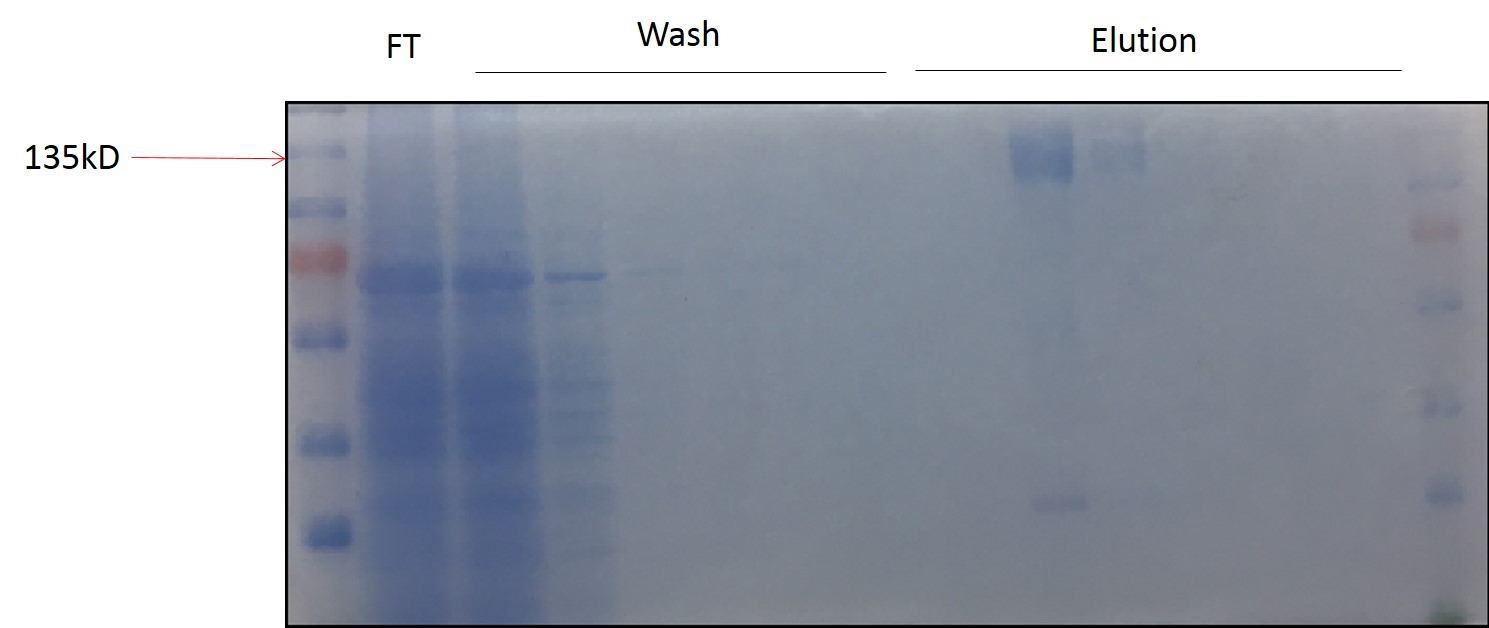
**

**Suppl. Fig. S1 Murine VISTA-Fc expression and purification.** (a) Protein expression in the cell supernatant gradually increased after transfection. (b) The protein was purified from the cell supernatant using Protein A resin. The murine VISTA-Fc protein was detected by Western blotting. (c) The purity of the recombinant VISTA-Fc protein was determined by SDS-PAGE.

**Suppl. Fig. S2a**


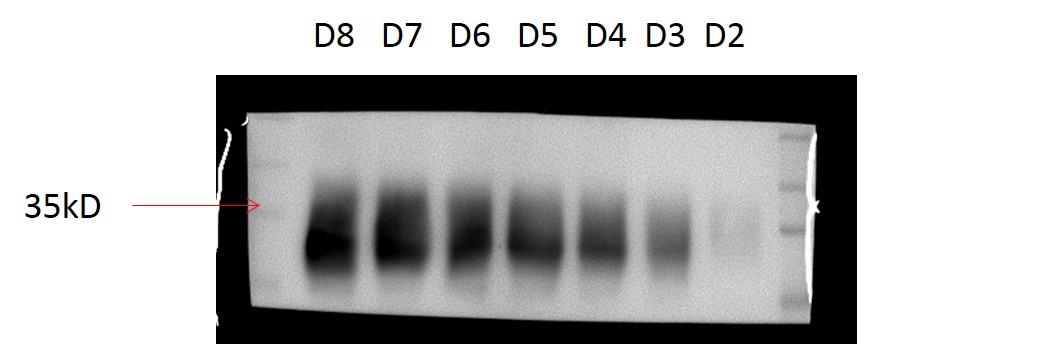


**Suppl. Fig. S2b**

**
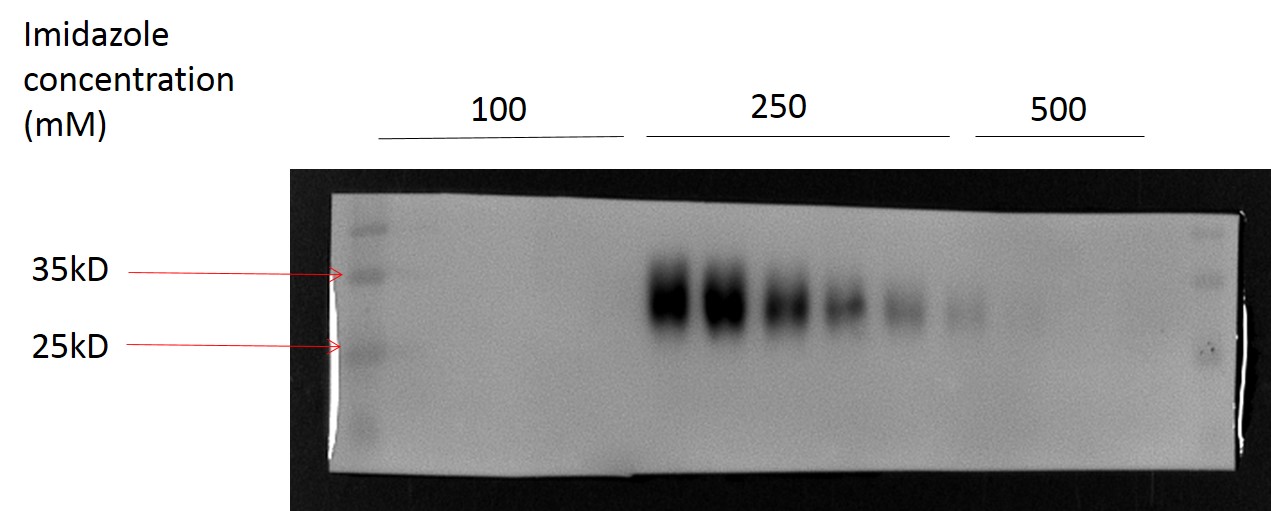
**

**Suppl. Fig. S2c**

**
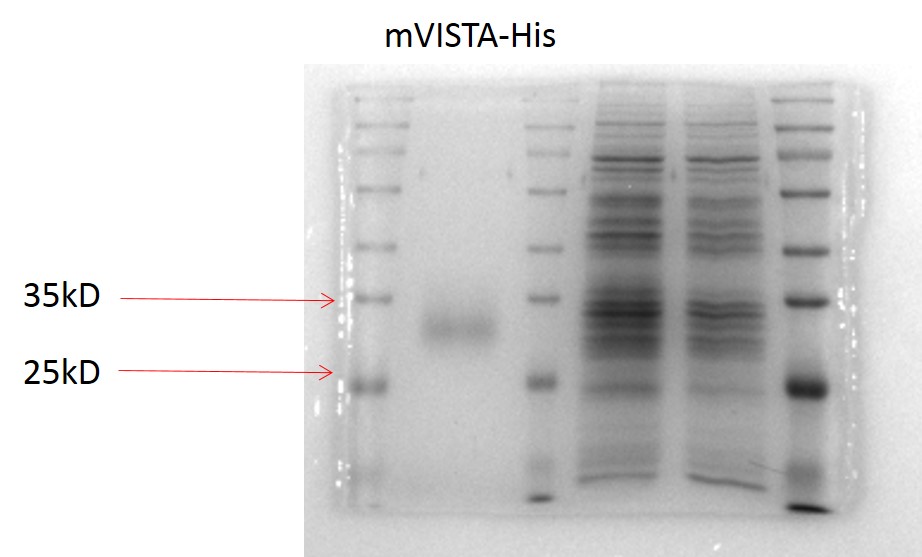
**

**Suppl. Fig. S2 Murine VISTA-His expression and purification.** (a) Protein expression in the cell supernatants as detected by Western blotting after transfection. (b) The protein was purified from the cell supernatant using Ni Sepharose^TM^ 6 Fast Flow resin. The murine VISTA-his10 protein was detected by Western blotting. (c) The purity of the recombinant VISTA-His protein was determined by SDS-PAGE.

**The binding rate of compounds and the VISTA-ECD protein**

The binding rates of murine VISTA-ECD with the compounds selected by virtual screening were evaluated with the ELISA assay (Suppl. Table S1).

**Suppl. Table S1** The binding rate of murine VISTA with compounds by ELISA assay

The absorbance was read at 450 nm (OD_450_) and 570nm(OD_570_).The absorbance at 570nm can be subtracted from the absorbance at 450nm. Binding rates(%)=(OD_compound_-OD_protein_)/OD_protein_×100%

| Number | Structure | Binding Rate（%） |
| --- | --- | --- |
| 6574-0488 |  | 9.12 |
| 6809-0223 |  | 16.02 |
| 6809-0226 |  | 14.03 |
| C176-0096 |  | 16.10 |
| C176-0112 |  | 8.59 |
| C176-0128 |  | 2.55 |
| C176-0202 |  | 3.73 |
| C206-0530 |  | 1.26 |
| C226-3957 |  | 5.90 |
| C594-0023 |  | 6.08 |
| F057-0346 |  | 3.35 |
| F057-0409 |  | 4.32 |
| F828-2036 |  | 3.35 |
| L471-0042 |  | 0.64 |
| L498-1241 |  | N/A |
| L822-0195 |  | N/A |
| L839-0030 |  | 6.43 |
| L922-0088 |  | 8.11 |
| L923-0517 |  | 5.49 |
| M321-0136 |  | 4.25 |
